# LC–MS/MS-guided discovery of japonamides C and D, two new cyclohexadepsipeptides, from the *Nicotiana tabacum*-derived endophytic fungus *Aspergillus japonicus* TE-739D

**DOI:** 10.3389/fmicb.2025.1595569

**Published:** 2025-05-08

**Authors:** Haisu Wang, Xianwei Hao, Chen Dong, Xiaolong Yuan, Peng Zhang, Gan Gu

**Affiliations:** ^1^Research Center for Plant Functional Components, Tobacco Research Institute of Chinese Academy of Agricultural Sciences, Qingdao, China; ^2^Technological Center, China Tobacco Zhejiang Industrial Co., Ltd., Hangzhou, China

**Keywords:** endophytic fungus, *Aspergillus japonicas*, cyclohexadepsipeptides, cell proliferation inhibitory activity, japonamides

## Abstract

Endophytic fungi belonging to the *Aspergillus* genus have received substantial attention due to their notable secondary metabolic potential. In this study, chemical investigations using LC–MS/MS-based molecular networking on the endophytic fungus *Aspergillus japonicus* TE-739D led to the discovery of two new cyclohexadepsipeptides, namely japonamides C (**1**) and D (**2**), along with three known cyclodipeptides (**3**–**5**). Their structures, including the absolute configurations of the amino acid residues, were elucidated through spectroscopic data analysis and an optimized Marfey’s method. The newly discovered compounds, japonamides C (**1**) and D (**2**), were screened for broad-spectrum cell proliferation inhibitory activity against 20 different human cell lines. The results indicated that both compounds displayed broad-spectrum antiproliferative activity against MKN-45, HCT116, TE-1, 5,637, CAL-62, and A-637 cells, with inhibition rates ranging from 55.0 to 72.3%. Moreover, the antibacterial activity of compounds **1**–**5** against two Gram-positive bacteria and two Gram-negative bacteria was also evaluated.

## 1 Introduction

Filamentous fungi belonging to the *Aspergillus genus* are widely distributed across both terrestrial and marine environments (Sanchez et al., 2012). Numerous species of *Aspergillus* have demonstrated the ability to produce a diverse array of secondary metabolites, including polyketides, sterols, fatty acids, peptides, alkaloids, terpenoids, and other miscellaneous compounds. These metabolites posses a wide range of biological activities, such as antimicrobial, cytotoxic, anti-inflammatory, herbicidal, and antioxidant properties (El-Hawary et al., 2020; Orfali et al., 2021). For instance, *A. ustus* has been identified as a source of three rearranged ergostane-type aspersteroids A–C, which exhibit significant immunosuppressive and antimicrobial activities (Liu et al., 2021). Moreover, the compound 12*β*,25,28-trihydroxyergone isolated from *A. terreus* has been shown to possess promising cytotoxic effects against the human colon cancer SW620 cell line and five human leukemia cell lines, with IC_50_ values ranging from 5.7 to 8.9 μM (Zhang et al., 2024). These results highlight the genus *Aspergillus* as a crucial source of novel secondary metabolites with biomedical potential.

Cyclic peptides are polypeptide chains composed of both canonical and non-canonical amino acids that are linked at distant positions to form macrocyclic structures. Current reports indicate that more than 40 cyclic peptide drugs are utilized in clinical settings, the majority of which are derived from natural products (NPs) or their derivatives (Zorzi et al., 2017; Zhang et al., 2021). For instance, commercial vancomycin, a heptapeptide composed of seven amino acids, exhibits strong antimicrobial efficacy against Gram-positive bacteria and is employed in the treatment of severe infections caused by methicillin-resistant *Staphylococcus aureus* (Walsh, 1999). In addition, cyclosporin A, which is a cyclic undecapeptide discovered in the 1970s, is recognized as a potent immunosuppressive agent (Ruegger et al., 1976; Ono et al., 2021). The significant biological activities and potential therapeutic applications of cyclic peptides have garnered considerable interest from both researchers and pharmaceutical companies. Consequently, there is an urgent need to identify new cyclic peptides with promising medicinal properties.

**Figure 1:**
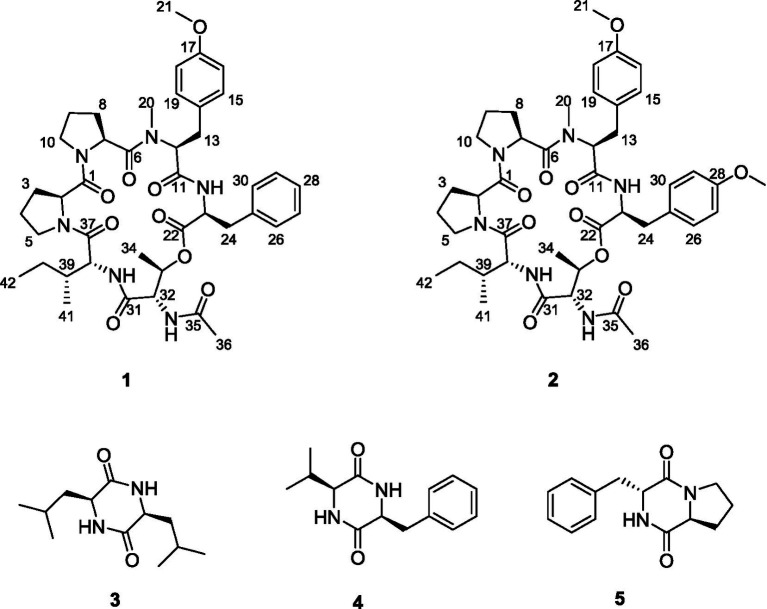
Structures of the isolated compounds **1**–**5**.

Traditional approaches for discovering novel cyclic peptides often result in a high rate of rediscovering known cyclic peptides. Global Natural Products Social Molecular Networking (GNPS; http://gnps.ucsd.edu) is an open-access knowledge base for community-wide organization and sharing of raw, processed, or annotated fragmentation mass spectrometry (MS/MS) data. It is widely utilized in the search for novel natural products (Wang et al., 2016; Zou et al., 2023; Cui et al., 2022). In our ongoing search for new bioactive metabolites from endophytic fungi (Gu et al., 2023; Gu et al., 2024a; Wang et al., 2025), we employed LC–MS/MS-based molecular networking for the rapid discovery of novel natural products (NPs) from the *Nicotiana tabacum*-derived endophytic fungus *A. japonicus* TE-739D. As a result, two new cyclohexadepsipeptides, japonamides C (**1**) and D (**2**), along with three known cyclodipeptides (**3**–**5**), were isolated and characterized (Figure 1). In addition, some of these compounds were evaluated for their antiproliferative and antibacterial activities.

## 2 Materials and methods

### 2.1 General experimental procedures

UV spectra were obtained using a Techcomp UV2310II spectrophotometer (Techcomp, Ltd., Shanghai, China). Specific rotations were recorded on a Rudolph Autopol IV automatic polarimeter (Rudolph Research Analytical, NJ, USA). Circular dichroism (CD) spectra were recorded on a JASCO J-1500 CD spectrometer (JASCO Corp., Tokyo, Japan). High-resolution electrospray ionization mass spectrometry (HRESIMS) spectra were recorded on a Micromass Q-TOF spectrometer (Waters, Milford, MA). ^1^H, ^13^C, and 2D NMR (HSQC, HMBC, ^1^H-^1^H COSY, NOESY) spectra were measured on a JNM-ECZ600R/S1 600 MHz NMR spectrometer (JEOL Ltd., Tokyo, Japan) and an Agilent DD2 500 MHz NMR spectrometer (Agilent Technologies, Santa Clara, CA). Chemical shifts were expressed in *δ* (ppm) relative to the solvent residual peaks at *δ*_H_ 2.50 and *δ*_C_ 39.5 for DMSO-*d*_6_, with coupling constants (*J*) reported in hertz (Hz). Column chromatography (CC) was performed using silica gel (100–200 mesh; Qing Dao Hai Yang Chemical Group Co., Qingdao, China), Sephadex LH-20 (GE Healthcare, Pittsburgh, PA), and reversed-phase (RP)-18 gel (40–60 μm, Merck, Darmstadt, Germany). Semipreparative HPLC separation was carried out on an NS4201 instrument (Hanbon Sci.& Tech., Jiangsu, China), equipped with an NP7000 SERIALS pump (flow rate: 3 ml/min) and an NU3000 SERIALS UV detector, using a SunFire-C_18_ column (150 mm × 10 mm i.d., 5 μm, Waters, Milford, MA).

### 2.2 Fungal source

The fungal strain *A. japonicus* TE-739D was isolated in 2018 from the leaves of cultivated tobacco (*N. tabacum* L.) grown in Enshi, Hubei Province (People’s Republic of China). Based on the phylogenetic analyses of the 28S and internal transcribed spacer (ITS) rDNA regions, this fungus was identified as *A. japonicus*, which has been deposited in GenBank (NCBI) under number PP126510. The strain was also deposited in the China General Microbiological Culture Collection Center (CGMCC No. 40901).

### 2.3 Fermentation, extraction, and isolation

The endophytic fungus *A. japonicus* TE-739D was statically cultivated in 290 × 1 L Erlenmeyer flasks containing 400 ml of potato dextrose water medium at 28°C for 30 days. Then, the fermentation product was extracted with ethyl acetate (EtOAc), and the crude extract (68.8 g) was obtained by evaporation under vacuum at 40°C. The extract was eluted using column chromatography on silica gel with stepwise petroleum ether (PE)–EtOAc mixtures (100:0, 90:10, 80:20, 70:30, 50:50, 30:70, and 0:100, v/v) to yield fractions Fr.1–Fr.6. Fr.6 was subjected to ODS CC with a MeOH–H_2_O gradient, (30:70–100:0, v/v) yielding seven fractions (Fr.6.1–Fr.6.7). Fr.6.6 was separated using Sephadex LH-20 CC (CH_2_Cl_2_-MeOH) to yield three subfractions (Fr.6.6.1–Fr.6.6.3). Compounds **1** (6.2 mg, *t*_R_ 32.042 min) and **2** (9.5 mg, *t*_R_ 34.542 min) were obtained from Fr.6.6.1 using semipreparative HPLC with MeCN–H_2_O (40:60, v/v). Compounds **3** (16.3 mg, *t*_R_ 11.045 min) and **4** (2.4 mg, *t*_R_ 13.345 min) were obtained from Fr.6.3 using semipreparative HPLC with MeCN–H_2_O (17:83, v/v). Similarly, compound **5** (6.2 mg, *t*_R_ 14.188 min) was obtained from Fr.6.3 using semipreparative HPLC with MeCN–H_2_O (22:78, v/v).

### 2.4 Spectral data of the isolated compounds

Japonamide C (**1**): Pale yellow powder; [*α*]^24^_D_–9.0 (*c* 0.30, MeOH); UV (MeOH) *λ*_max_ 200, 226, 275 nm; ^1^H NMR (DMSO-*d*_6_, 500 MHz), ^13^C NMR (DMSO-*d*_6_, 125 MHz) (see Table 1); HRESIMS *m/z* 789.4180 [M + H]^+^ (calcd. For C_42_H_57_N_6_O_9_, 789.4182).

**Table 1:** ^1^H NMR data (500 MHz) and ^13^C NMR data (125 MHz) for compounds 1 and 2.

Position	**1** (DMSO-*d*_6_)		**2** (DMSO-*d*_6_)	
	*δ*_H_ mult. (*J* in Hz)	*δ*_C_ type	*δ*_H_ mult. (*J* in Hz)	*δ*_C_ type
	**L-Pro-1**		**L-Pro-1**	
1		169.7 C		169.7 C
2	4.63 dd (8.8, 3.5)	57.3 CH	4.62 dd (8.8, 3.5)	57.3 CH
3	2.17 m 1.73 m	27.4 CH_2_	2.16 dd (12.8, 8.8) 1.71 m	27.4 CH_2_
4	1.88 m	24.0 CH_2_	1.88 m	24.0 CH_2_
5	3.85 td (8.9, 5.5) 3.62 m	47.1 CH_2_	3.82 m 3.58 m	47.1 CH_2_
	**L-Pro-2**		**L-Pro-2**	
6		172.2 C		172.2 C
7	4.32 m	54.4 CH	4.33dd (8.1, 5.5)	54.5 CH
8	1.02 m 0.72 m	28.1 CH_2_	1.02 m 0.72 dt (12.7, 6.5)	28.0 CH_2_
9	1.88 m 1.62 m	25.0 CH_2_	1.88 m 1.63 m	25.0 CH_2_
10	3.65 m 3.59 m	46.8 CH_2_	3.65 m 3.44 m	46.8 CH_2_
	***N*-Me-*O*-Me-D-Tyr**		***N*-Me-*O*-Me-D-Tyr**	
11		169.3 C		169.3 C
12	4.80 dd (11.5, 3.6)	61.7 CH	4.81 dd (11.5, 3.6)	61.7 CH
13	2.98 m 2.71 dd (14.5, 11.5)	32.2 CH_2_	2.98 dd (14.4, 3.6) 2.73 dd (14.4, 11.5)	32.1 CH_2_
14		129.7 C		129.7 C
15 or 19	7.10 d (8.5)	130.4 CH	7.10 d (8.6)	130.4 CH
16 or 18	6.84 d (8.5)	113.9 CH	6.84 d (8.6)	113.9 CH
17		158.0 C		158.1 C
20	2.14 s	28.0 CH_3_	2.21 s	28.1 CH_3_
21	3.70 s	55.1 CH_3_	3.70 s	55.1 CH_3_
	**L-Phe**		***O*-Me-L-Tyr**	
22		170.5 C		170.6 C
23	4.35 m	55.4 CH	4.28 td (8.4, 5.7)	55.7 CH
24	2.97 m	37.0 CH_2_	2.88 m	36.3 CH_2_
25		137.7 C		129.6 C
26 or 30	7.18 m	129.1 CH	7.09 d (8.6)	130.2 CH
27 or 29	7.23 m	128.3 CH	6.78 d (8.6)	113.7 CH
28	7.21 m	126.4 CH		157.9 C
28-OCH_3_			3.70 s	55.0 CH_3_
NH	8.70 d (8.5)		8.68 d (8.4)	
	***N*-Ac-L-Thr**		***N*-Ac-L-Thr**	
31		167.7 C		167.7 C
32	4.73 dd (9.1, 4.5)	54.5 CH	4.71 dd (9.1, 4.5)	54.5 CH
33	4.97 qd (6.5, 4.2)	70.0 CH	4.97 m	70.0 CH
34	1.22 d (6.7)	15.1 CH_3_	1.21 d (6.7)	15.2 CH_3_
35		170.0 C		170.0 C
36	1.98 s	22.7 CH_3_	1.99 s	22.7 CH_3_
NH	7.82 d (9.1)		7.82 d (9.1)	
	**D-Ile**		**D-Ile**	
37		169.8 C		169.9 C
38	4.42 t (8.9)	54.5 CH	4.42 t (8.9)	54.5 CH
39	1.71 m	36.3 CH	1.71 m	36.2 CH
40	1.32 m 1.07 m	25.1 CH_2_	1.31 m 1.07 m	25.2 CH_2_
41	0.82 d (6.7)	14.7 CH_3_	0.82 d (6.7)	14.7 CH_3_
42	0.85 t (7.4)	11.6 CH_3_	0.85 t (7.4)	11.6 CH_3_
NH	7.38 d (8.9)		7.36 d (8.9)	

Japonamide D (**2**): Pale yellow powder; [*α*]^24^_D_ + 106.7 (c 0.24, MeOH); UV (MeOH) *λ*_max_ 200, 228, 280 nm; ^1^H NMR (DMSO-*d*_6_, 500 MHz), ^13^C NMR (DMSO-*d*_6_, 125 MHz) (see Table 1); HRESIMS *m/z* 819.4294 [M + H]^+^ (calcd. For C_43_H_59_N_6_O_10_, 819.4287).

### 2.5 Absolute configurations of amino acids using advanced Marfey’s analysis

Optimized Marfey’s analyses were carried out as previously reported (Wang et al., 2022). Compounds **1** and **2** (1.0 mg) were dissolved in 6 M HCl (1.0 ml) and heated at 100°C for 24 h. The solutions were then evaporated to dryness, transferred to a 4-ml reaction vial, and treated with a 10-mg/ml solution of 1-fluoro-2-4-dinitrophenyl-5-L-alanine amide (FDAA, 200 μl) in acetone, followed by 1.0 M NaHCO_3_ (40 μl). The reaction mixtures were heated at 45°C for 90 min, and the reactions were quenched by the addition of HCl (1 M, 40 μl). Similarly, standard L-and D-amino acids (Pro, *N*-Me-*O*-Me-Tyr, Phe, Thr, Ile, *O*-Me-Tyr) were derivatized separately. The derivatives of the acid hydrolysate and the standard amino acids were analyzed using HPLC (ACQUITY C_18_ column; 1.7 μm, 2.1 × 100 mm; 0.3 ml/min; UV detection at 340 nm), with a linear gradient of acetonitrile (30–45%) in water (TFA, 0.01%) for 20 min. Retention times for the authentic standards were as follows: L-Thr (1.85 min), D-Thr (2.35 min), L-Pro (2.88 min), D-Pro (3.25 min), FDAA (3.54 min), L-Ile (6.77 min), *N*-Me-*O*-Me-L-Tyr (6.91 min), *O*-Me-L-Tyr (7.15 min), L-Phe (7.24 min), *O*-Me-D-Tyr (9.70 min), D-Phe (10.03 min), and D-Ile (10.48 min). The absolute configurations of the chiral amino acids in compounds **1** and **2** were determined by comparing the retention times.

### 2.6 Cell proliferation inhibitory activity

The antiproliferative activity of the compounds was tested against 18 human cancer cell lines—the lung cancer cell line A549, stomach cancer cell line MKN-45, colon cancer cell line HCT 116, cervical cancer cell line HeLa, chronic myelogenous leukemia cell line lK-562, renal clear cell adenocarcinoma cell line 786-O, esophageal cancer cell line TE-1, bladder cancer cell line 5,637, gallbladder cancer cell line GBC-SD, breast cancer cell line MCF7, liver cancer cell line HepG2, brain tumor cell line SF126, prostate cancer cell line DU145, thyroid cancer cell line CAL-62, pancreatic cancer cell line PATU8988T, osteosarcoma cell line HOS, malignant melanocyte cell line A-375, and rhabdomyosarcoma cell line A-673. In addition, two non-cancerous cell lines—the human normal liver cell line L-02 and human embryonic kidney cell line 293 T—were also included. The Cell Counting Kit-8 (CCK-8) was used to evaluate the proliferation inhibitory activity of japonamides C (**1**) and D (**2**), as described previously (Xu et al., 2014; Wu et al., 2015). Cisplatin was used as a positive control.

### 2.7 Antibacterial assays

The antibacterial activity of compounds **1**–**5** was evaluated against two Gram-positive bacteria—*Bacillus cereus* and *B. subtilis*—and two Gram-negative bacteria—*Ralstonia solanacearum* and *Xanthomonas oryzae*. Streptomycin sulfate was used as a positive control. The minimum inhibitory concentrations (MICs) of each compound were determined using the broth micro-dilution method with certain modifications (Song et al., 2021; Gu et al., 2024b).

### 2.8 UHPLC-Q-TOF and molecular networking analysis

The extract of the fungus *A. japonicus* TE-739D was dissolved in MeOH (10 mg/ml) and then analyzed using LC–MS/MS. The mobile phase consisted of 0.1% formic acid in water (A) and 0.1% formic acid in MeCN (B). The elution started with a 5% solvent B isocratic phase for 5 min, followed by a linear gradient from 5 to 95% solvent B over 5 to 35 min and then maintained at 95% solvent B for 5 min. The flow rate was 0.3 ml/min, and the injection volume was 5 μl. The MS survey scan covered a range of 200–2,000 *m/z*, and the MS/MS scan ranged from 100 to 1,000 *m/z*. The raw MS/MS data were converted to mzML files using the MSConvert 3.0 software, and the data were then submitted to the GNPS platform (https://gnps.ucsd.edu, accessed on 1 January 2016). The parameters for molecular network generation were set as follows: Precursor ion mass tolerance set to 0.02 Da, fragment ion mass tolerance at 0.02 Da, min pairs cos at 0.6, a minimum of 5 matched fragment ions, maximum shift of 600, a minimum cluster size of 2, and a network TopK value of 10. The generated molecular network was visualized using Cytoscape 3.8.2.

## 3 Results

### 3.1 Molecular networking analysis for the discovery of new cyclopeptides from *A. japonicas* TE-739D

**Figure 2:**
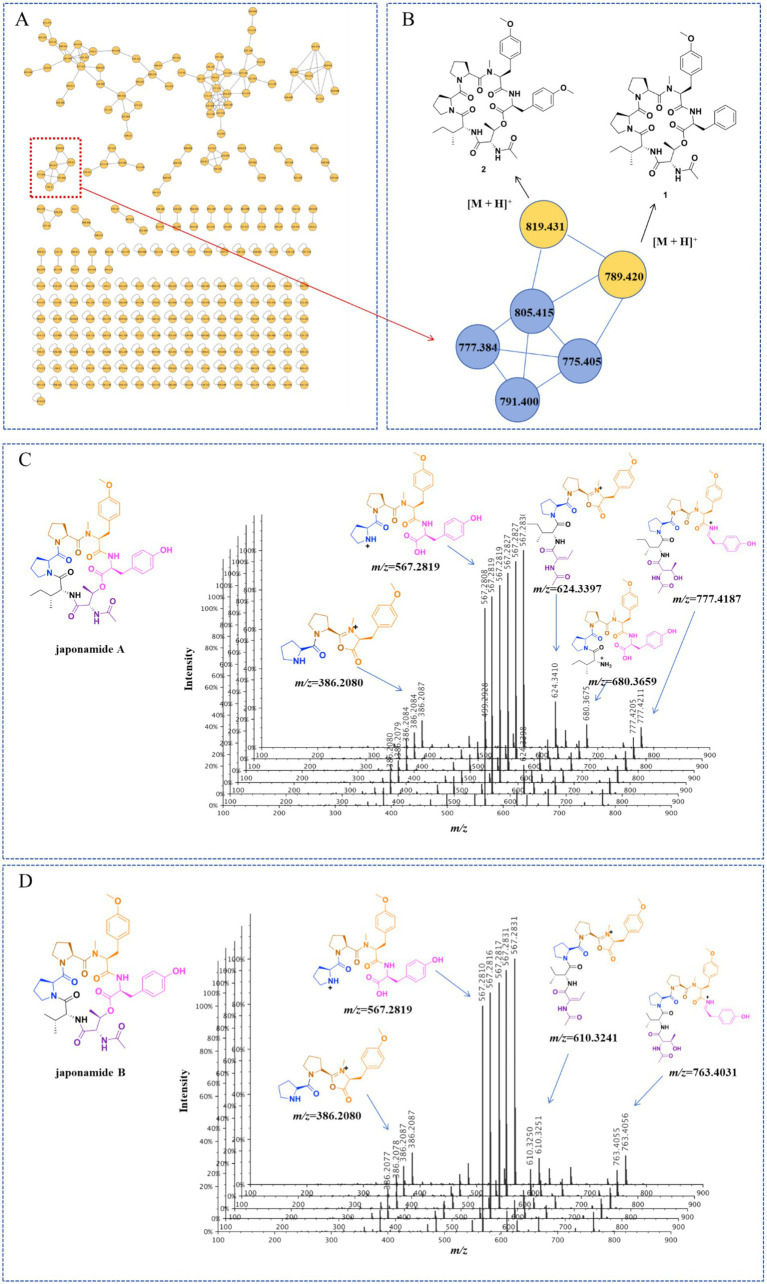
Molecular networking-based prioritization of the EtOAc extract of *A. japonicus* TE-739D for peptide natural products: **(A)** complete network; **(B)** cluster of peptides including compounds **1** and **2**; tandem mass spectrometry of protonated ions at *m/z* 791.400 **(C)** and 805.415 **(D)**, showing typical amino acid imine ion fragments of japonamides A and B.

Following the above-mentioned extraction and concentration procedures, a total of 68.8 g of ethyl acetate (EtOAc) extract was obtained from 116 L of the PDB fermentation broth derived from *A. japonicus* TE-739D. This extract was analyzed using high-resolution tandem mass spectrometry (HR-MS/MS). Subsequently, a molecular network (MN) was constructed utilizing the GNPS platform (Figure 2A). The analysis revealed the presence of more than 13 prominent clusters. One particular cluster within this molecular network comprised precursor ions in the *m/z* range of 700–900 (Figure 2B), which were highly similar to the cluster reported in the literature (Wang et al., 2022). The MS/MS spectrum of two nodes with protonated ions at *m/z* 791.400 and 805.415 [M + H]^+^ matched the cyclohexadepsipeptides japonamide A (Figure 2C) and japonamide B (Figure 2D). The presence of surrounding nodes with similar masses within this cluster suggested that *A. japonicus* TE-739D may possess unique potential to produce novel cyclohexadepsipeptide analogs.

### 3.2 Structure elucidation of the new compounds

Compound **1** was obtained as a pale yellow powder. The HRESIMS data (Supplementary Figure S8) revealed a protonated molecule at *m/z* 789.4185 (calculated value: 789.4182), which was consistent with the molecular formula C_42_H_56_N_6_O_9_, implying 18 degrees of unsaturation. The ^1^H NMR data (Table 1) and HSQC spectrum of compound **1** showed signals for three amide protons [δ_H_ 8.70 (1H, d, J = 8.5 Hz), 7.82 (1H, d, J = 9.1 Hz), 7.38 (1H, d, J = 8.9 Hz)], a monosubstituted phenyl group (δ_H_ 7.23, 7.23, 7.21, 7.18, and 7.18), a 1,4-disubstituted phenyl group (δ_H_ 7.10, 7.10, 6.84, and 6.84), seven oxygenated or nitrogenated methine protons (δ_H_ 4.97, 4.80, 4.73, 4.63, 4.42, 4.35, and 4.32), two oxygenated or nitrogenated methylene protons (δ_H_ 3.85/3.62, 3.65/3.59), one O-methyl group (δ_H_ 3.70), one N-methyl group (δ_H_ 2.14), four methyl groups (δ_H_ 1.98, 1.22, 0.85, and 0.82), and several aliphatic protons. The ^13^C NMR data (Table 1), combined with the HSQC spectra, revealed 38 carbon resonance signals, including 7 carbonyl compounds (δ_C_ 172.2, 170.5, 170.0, 169.8, 169.7, 169.3, and 167.7), 12 olefinic carbons including four superimposable ones (δ_C_ 113.9 and 130.4), 9 oxygenated or nitrogenated carbons (δ_C_ 70.0, 61.7, 57.3, 55.4, 54.5, 54.5, 54.4, 47.1, and 46.8), 6 methyl carbons including one methoxy (δ_C_ 55.1), and 8 aliphatic carbons (δ_C_ 37.0, 36.3, 32.2, 28.1, 27.4, 25.1, 25.0, and 24.0).

**Figure 3:**
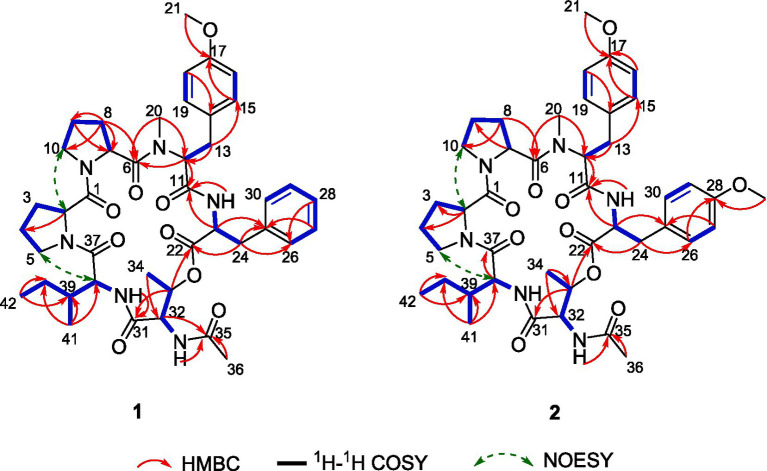
Key ^1^H–^1^H COSY, HMBC, and NOESY correlations of compounds **1** and **2**.

Further analysis of the 2D NMR spectra (Supplementary Figures S3–S5) led to the identification of six amino acid components. The first aliphatic amino acid (Pro-1) was confirmed by the ^1^H–^1^H COSY correlations of H-2/H_2_-3/H_2_-4/H_2_-5 and the HMBC correlations from H-2 to C-4 (Figure 3). Another proline (Pro-2) residue was constructed with similar resonance signals (Figure 3). An N,O-dimethyl-tyrosine unit (N-Me-O-Me-Tyr) was confirmed by the HMBC correlations from H_3_-20 to C-11, from H-12 to C-6/C-11/C-20, from H_2_-13 to C-12/C-15, from H-15 to C-13/C-17, from H-16 to C-14, and from H_3_-21 to C-17, as well as by the ^1^H–^1^H COSY correlations between H-2/H_2_-3, H-15/H-16, and H-18/H-19 (Figure 3). In addition, a spin system consisting of five adjacent aromatic protons (δ_H_ 7.23, 7.23, 7.21, 7.21, and 7.18), recognized in the ^1^H–^1^H COSY correlations (Figure 3), was identified as a monosubstituted phenyl group. The HMBC correlations from H-23 to C-22/C-24/C-25 and from H_2_-24 to C-22/C-23/C-25/C-26 indicated the presence of a phenylalanine unit (Phe). The N-acetyl-threonine residue (N-Ac-Thr) was confirmed by the ^1^H–^1^H COSY correlations of H-32/H-33/H_3_-34 and the HMBC correlations from H-32 to C-31/C-33/C-35, from H-33 to C-31/C-34, from H_3_-34 to C-32/C-33, and from H_3_-36 to C-35. The last unit was identified as isoleucine (Ile) by the ^1^H–^1^H COSY correlations of H-38/H-39/H_2_-40/H_3_-41, H_2_-40/H_3_-42, as well as the HMBC correlations from H_3_-41 to C-38/C-39/C-40 and from H_3_-42 to C-39/C-40.

The connectivity among the six amino acid residues was determined as Pro-Pro-(N-Me-O-Me-Tyr)-Phe-(N-Ac-Thr)-Ile by the NOESY correlations of H-2/H_a_-10 (δ_H_ 3.65) and the HMBC correlations from H_3_-20 to C-6, from H-23 to C-11, from H-33 to C-22, and from Ile-NH to C-31 (Figure 3). The peptide chain was closed by the NOESY correlations between H_2_-5 and H-38. Two proline residues were confirmed by the small chemical shift difference between C-3/C-4 (δ_C-3_–_C-4_ = 3.4 ppm) in Pro-1 and C-8/C-9 (δ_C-8_–_C-9_ = 3.1 ppm) in Pro-2, observed in the ^13^C NMR spectrum (Supplementary Figure S2), which are consistent with the presence of a trans proline (Siemion et al., 1975; Li et al., 2024).

**Scheme 1:**
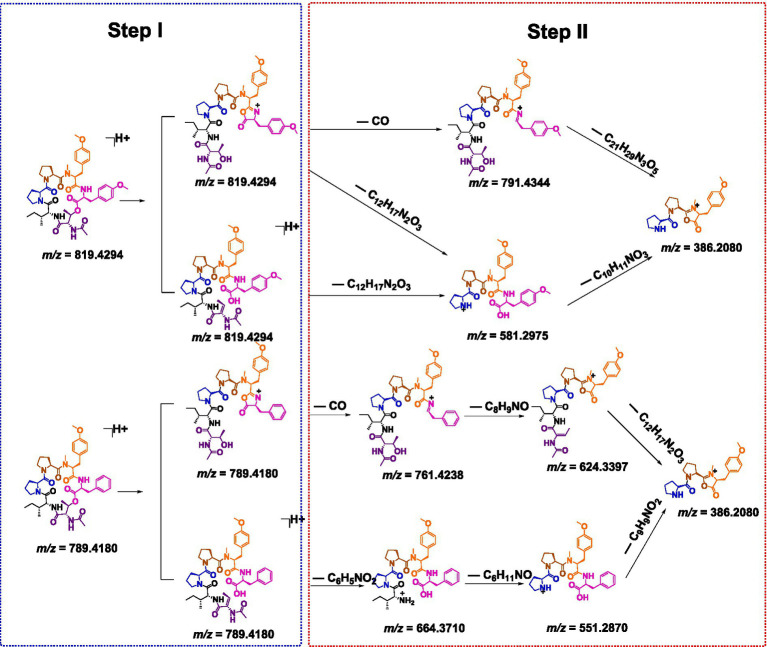
Plausible formation mechanism for the MS/MS ion fragments of compounds **1** and **2**.

Tandem mass spectrometry (MS/MS) enables the precise determination of amino acid sequences by leveraging diagnostic fragmentation patterns under controlled collision energies. Under low-energy collision-induced dissociation (CID), protonated peptides predominantly undergo amide bond cleavage, generating characteristic N-terminal (bn), C-terminal (y_n_), and a-type (b_n_–CO) ions, which are critical for sequence elucidation (Wang et al., 2022; Polce et al., 2000). As detailed in Scheme 1 and Supplementary Figure S17, compound **1** initially underwent lactone ring opening to yield a linear intermediate (Step I), followed by sequential elimination of amino acid residues via two distinct fragmentation pathways (Step II). The observed cleavage patterns closely resembled the patterns of the structurally characterized reference compound japonamide A (Wang et al., 2022), thereby corroborating both the fragmentation mechanism and the derived amino acid sequences.

**Figure 4:**
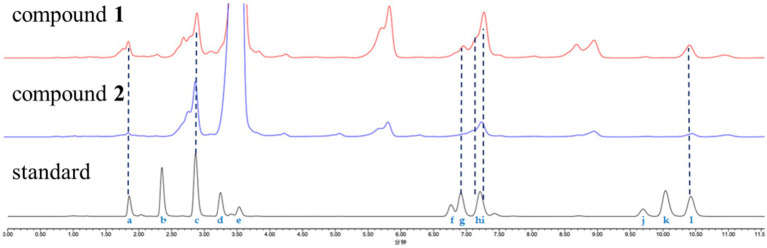
Advanced Marfey’s analysis of compounds **1** and **2**. The FDAA derivatives of the standard compounds a–l were as follows: L-Thr (1.85 min), D-Thr (2.35 min), L-Pro (2.88 min), D-Pro (3.25 min), FDAA (3.54 min), L-Ile (6.77 min), *N*-Me-*O*-Me-L-Tyr (6.91 min), *O*-Me-L-Tyr (7.15 min), L-Phe (7.24 min), *O*-Me-D-Tyr (9.70 min), D-Phe (10.03 min), and D-Ile (10.48 min). The derivatives of the acid hydrolysate and standard amino acids were analyzed using HPLC (ACQUITY C_18_ column; 1.7 μm, 2.1 × 100 mm; 0.3 ml/min; UV detection at 340 nm) with a linear gradient of acetonitrile (30–45%) in water (TFA, 0.01%) for 20 min.

The absolute configurations of the amino acid residues in compound **1** were unambiguously determined using an optimized Marfey’s method (Wang et al., 2022), involving acid hydrolysis, chiral derivatization with FDAA, and reversed-phase HPLC comparison with authentic L/D standards (Figure 4). The targeted acid hydrolysis (6 M HCl, 100°C) of compound **1**, followed by FDAA derivatization, revealed characteristic adducts for Pro, N-methyl-O-methyl-Tyr, Phe, Thr (from N-Ac-Thr hydrolysis), and Ile. The comparative HPLC analysis with authentic standards confirmed the configurations as L-Pro (×2), N-Me-O-Me-L-Tyr, L-Phe, N-Ac-L-Thr, and D-Ile, establishing the complete sequence as cyclo-(L-Pro)-(L-Pro)-(N-Me-O-Me-L-Tyr)-(L-Phe)-(N-Ac-L-Thr)-(D-Ile).

Compound **2** was obtained as a pale yellow powder. The molecular formula C_43_H_58_N_6_O_10_ was deduced from a positive HRESIMS ion at m/z [M + H]^+^ 819.4287 (calculated value: 819.4287), with 18 degrees of unsaturation. The ^1^H and ^13^C NMR data (Table 1) of compound **2** closely resembled those of compound **1**, indicating that they were structurally similar. The main difference was the absence of an aromatic proton in compound **2**, which was instead substituted by a methoxy group to form a 1,4-disubstituted phenyl group. The ^1^H–^1^H COSY correlations of H-26/H-27 and H-29/H-30, as well as the HMBC correlations from 28-OCH_3_ to C-28 and from H-26 to C-28, unequivocally supported this assignment (Figure 3). The remaining similar NMR spectra could establish the planar structure of compound **2**, as shown in Figure 3. Subsequent MS/MS analysis and Marfey’s analysis (Scheme 1; Supplementary Figure S17; Figure 4) established the structure of compound **2** as cyclo-(L-Pro)-(L-Pro)-(N-Me-O-Me-L-Tyr)-(O-Me-L-Tyr)-(N-Ac-L-Thr)-(D-Ile).

The known compounds were identified based on the literature data as cis-cyclo(Leu-Leu) (**3**) (He et al., 2013), trans-cyclo(Pro-Phe) (**4**) (Wang et al., 2010), and cis-cyclo(Phe-Val) (**5**) (Harizani et al., 2020).

### 3.3 Cell proliferation inhibitory activity and antibacterial activity

**Figure 5:**
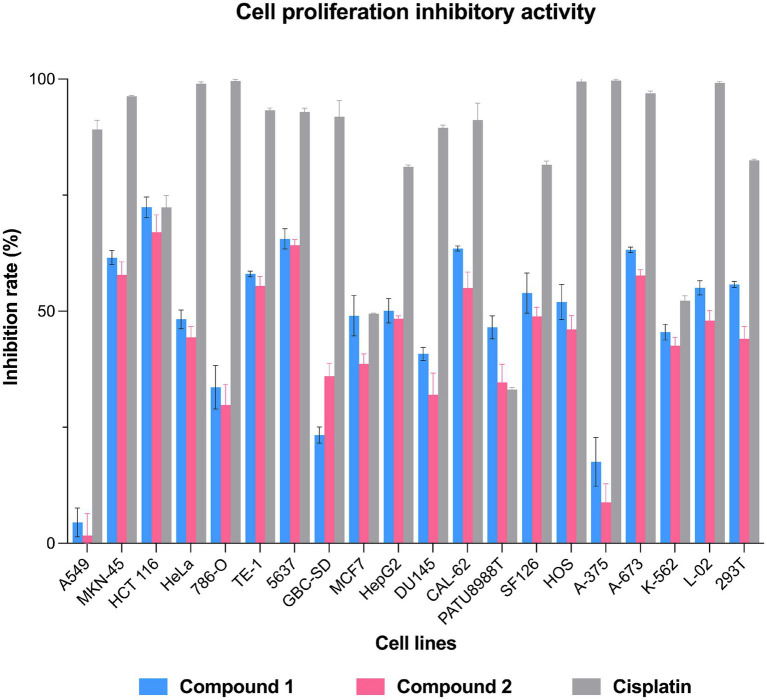
Cell inhibition rates of compounds **1** and **2** against the lung cancer cell line A549, stomach cancer cell line MKN-45, colon cancer cell line HCT 116, cervical cancer cell line HeLa, renal clear cell adenocarcinoma cell line 786-O, esophageal cancer cell line TE-1, bladder cancer cell line 5,637, gallbladder cancer cell line GBC-SD, breast cancer cell line MCF7, liver cancer cell line HepG2, prostate cancer cell line DU145, thyroid cancer cell line CAL-62, pancreatic cancer cell line PATU8988T, brain tumor cell line SF126, osteosarcoma cell line HOS, malignant melanocyte cell line A-375, rhabdomyosarcoma cell line A-673, chronic myeloid leukemia cell line K-562, human normal liver cell line L-02, and embryonic kidney cell line 293 T. Cisplatin was used as a positive control.

The antiproliferative activity of the two isolated cyclohexadepsipeptides, compounds **1** and **2,** was evaluated against 20 different cell lines, including 18 human tumor cell lines and 2 human normal cell lines. Both compounds showed broad-spectrum antiproliferative activity against MKN-45, HCT116, TE-1, 5,637, CAL-62, and A-637 cells at 20 μM, with inhibition rates ranging from 55.0 to 72.3% (Figure 5). No detectable cytotoxicity was observed for the two tested compounds against A549 and A-375 cells, indicating the selective cytotoxicity of the test compounds. In general, compound **1** exhibited superior cytotoxic activity compared to compound **2**. The available data from structure–activity relationship (SAR) analysis suggest that the C-28 substituent may serve as a critical pharmacophore for cytotoxic effects.

The antibacterial activity of compounds **1**–**5** was evaluated against four bacterial strains: *Ralstonia solanacearum* (G^−^), *Xanthomonas oryzae* (G^−^), *Bacillus cereus* (G^+^), and *Bacillus subtilis* (G^+^). As presented in Table 2, compounds **4** and **5** displayed only weak antibacterial activity against the two Gram-positive bacteria *B. cereus* and *B. subtilis*.

**Table 2:** Cytotoxic results of compounds 1–6 against four human tumor cell lines.

Compound	*R. solanacearum*	*X. oryzae*	*B. cereus*	*B. subtilis*
**1**	>128	>128	>128	>128
**2**	>128	>128	>128	>128
**3**	>128	>128	>128	>128
**4**	>128	>128	32	16
**5**	>128	>128	16	16
Streptomycin sulfate^a^	16	64	4	2

^*^Positive control.

## 4 Conclusion

In summary, this study reports the LC–MS/MS-guided isolation and structural characterization of two novel cyclohexadepsipeptides, japonamides C (**1**) and D (**2**), along with three known cyclodipeptides (**3**–**5**), from the *Nicotiana tabacum*-derived endophytic fungus *A. japonicus* TE-739D. The planar structures were unequivocally established through comprehensive 1D/2D NMR analysis and HRESIMS data, while the absolute configurations were resolved using an optimized Marfey’s protocol. A plausible formation mechanism for the MS/MS ion fragments of compounds **1** and **2** was proposed. In the broad-spectrum screening for antiproliferative activity, compounds **1** and **2** displayed moderate cytotoxicity against selected cancer cell lines. The observed SAR trends suggest a possible functional significance of the C-28 substituent in cytotoxicity, although further validation is needed. The results not only expand the chemical diversity of fungal-derived cyclopeptides with bioactivity but also exemplify the utilization of LC–MS/MS-based GNPS in accelerating the discovery of natural products from underexplored microbial sources.

## Data Availability

The datasets presented in this study can be found in online repositories. The names of the repository/repositories and accession number(s) can be found in the article/Supplementary material.
