# Supplementary material for: LC–MS/MS-guided discovery of japonamides C and D, two new cyclohexadepsipeptides, from the Nicotiana tabacum-derived endophytic fungus Aspergillus japonicus TE-739D
Source: Front Microbiol. 2025 May 8;16:1595569. doi: 10.3389/fmicb.2025.1595569 (PMC12095196; doi:10.3389/fmicb.2025.1595569)
Supplement: Supplementary file 1 [file Supplementary_file_1.docx]

**Supplementary Material**

**LC-MS/MS Guided Discovery of Japonamides C and D, Two New Cyclohexadepsipeptides from the *Nicotiana tabacum*-Derived Endophytic Fungus *Aspergillus japonicus* TE-739D**

Haisu Wang ^1,†^, Xianwei Hao ^2,†^, Chen Dong ^1^, Xiaolong Yuan ^1^, Peng Zhang ^1^ and Gan Gu ^1,^*

^1^Tobacco Research Institute of Chinese Academy of Agricultural Sciences, Qingdao 266101, China

^2^Technological Center, China Tobacco Zhejiang Industrial Co., Ltd., Hangzhou 310008, China

**^†^**These authors contributed equally to this work.

***Correspondence:** gugan@caas.cn (G. G.)

**Keywords:** endophytic fungus; *Aspergillus japonicas*; cyclohexadepsipeptides; cell proliferation inhibitory activity; japonamides

**Table of Contents**

[**Figure S1.** ^1^H NMR spectrum of **1** (DMSO-*d*_6_, 500 MHz) 3](#_Toc17355)

[**Figure S2.** ^13^C NMR spectrum of **1** (DMSO-*d*_6_, 125 MHz) 3](#_Toc25939)

[**Figure S3.** HSQC spectrum of **1** 4](#_Toc20770)

[**Figure S4.** ^1^H-^1^H COSY spectrum of **1** 4](#_Toc30191)

[**Figure S5.** HMBC spectrum of **1** 5](#_Toc651)

[**Figure S6.** NOESY spectrum of **1** 5](#_Toc4251)

[**Figure S7.** UV spectrum of **1** (100% MeOH/H_2_O, extracted from HPLC-DAD data) 6](#_Toc7156)

[**Figure S8.** HRESIMS spectrum of **1** 6](#_Toc9663)

[**Figure S9.** ^1^H NMR spectrum of **2** (DMSO-*d*_6_, 500 MHz) 7](#_Toc24457)

[**Figure S10.** ^13^C NMR spectrum of **2** (DMSO-*d*_6_, 150 MHz) 7](#_Toc23705)

[**Figure S11.** HSQC spectrum of **2** 8](#_Toc5712)

[**Figure S12.** ^1^H-^1^H COSY spectrum of **2** 8](#_Toc1515)

[**Figure S13.** HMBC spectrum of **2** 9](#_Toc4742)

[**Figure S14.** NOESY spectrum of **2** 9](#_Toc1371)

[**Figure S15.** UV spectrum of **2** (100% MeOH/H_2_O, extracted from HPLC-DAD data) 10](#_Toc8730)

[**Figure S16.** HRESIMS spectrum of **2** 10](#_Toc28309)

[**Figure S17.** The tandem mass spectrometry of protonated ions at m/z 789.420 (**A**) and 819.431 (**B**), showing typical amino acid imine ions fragments of japonamides C (**1**) and D (**2**). 11](#_Toc22961)


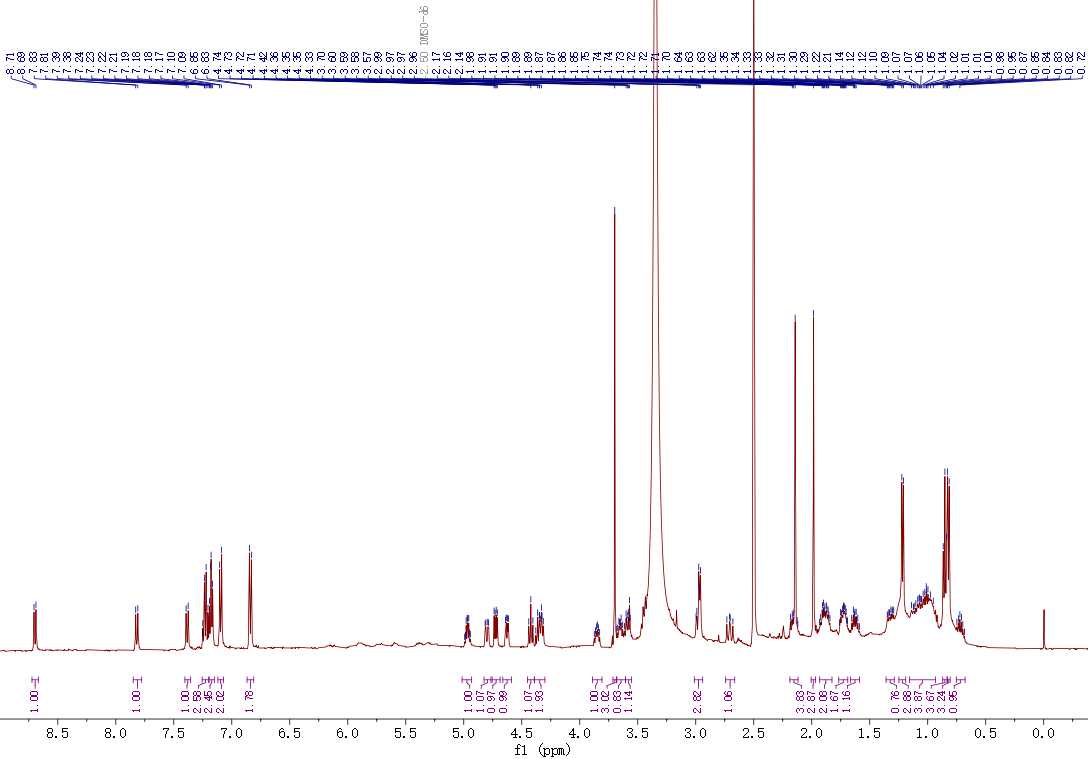


**Figure S1.** ^1^H NMR spectrum of **1** (DMSO-*d*_6_, 500 MHz)


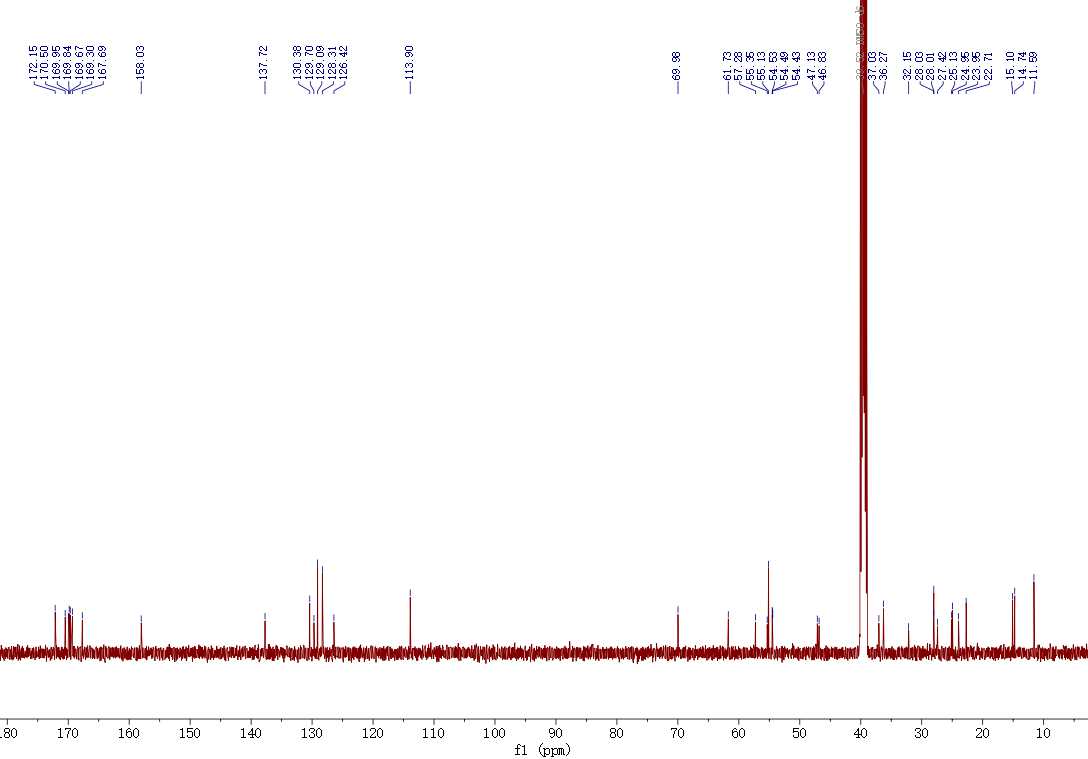


**Figure S2.** ^13^C NMR spectrum of **1** (DMSO-*d*_6_, 125 MHz)


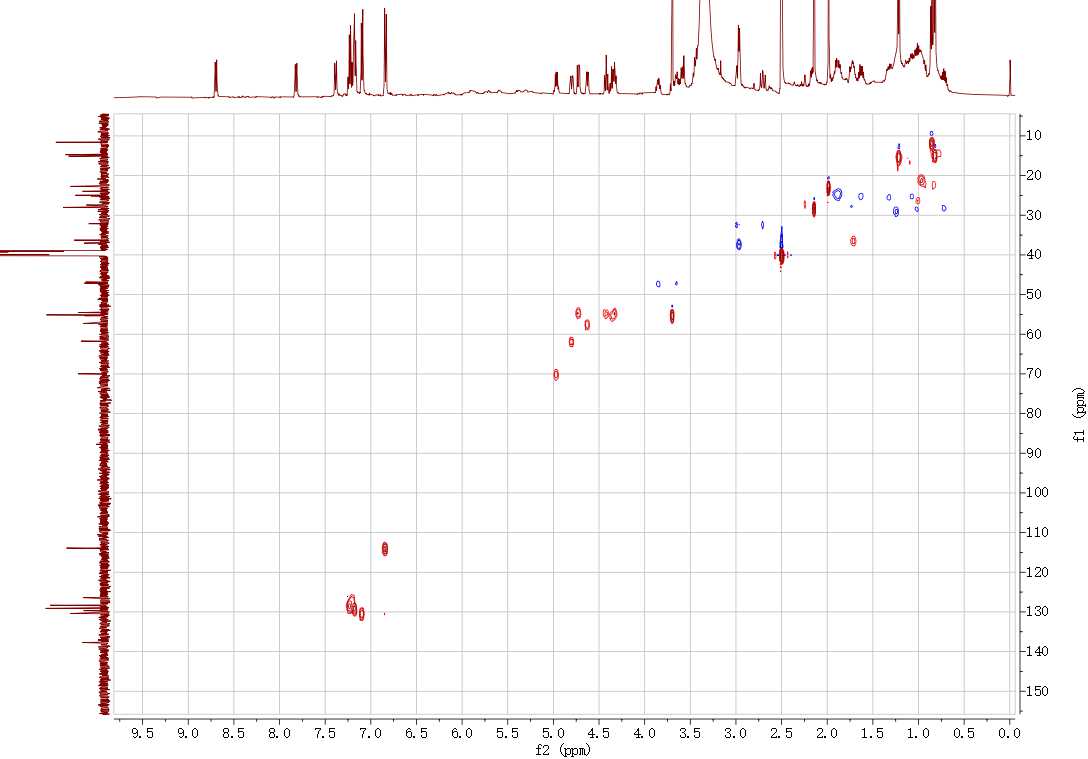


**Figure S3.** HSQC spectrum of **1**

**
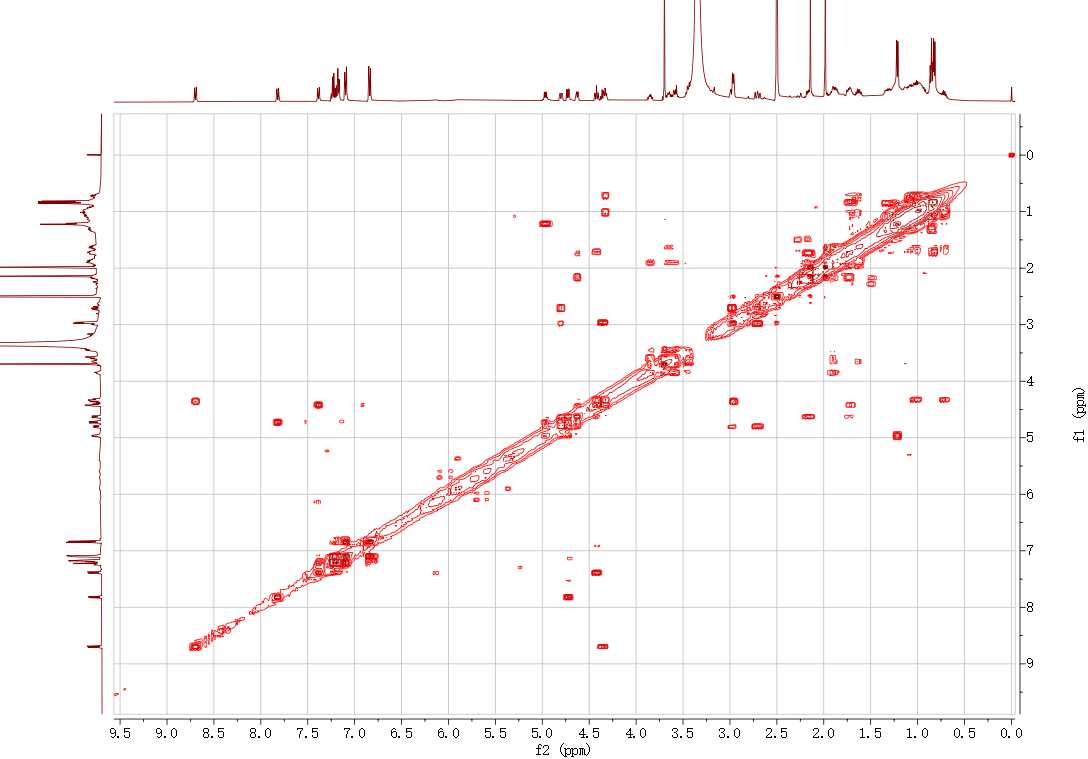
**

**Figure S4.** ^1^H-^1^H COSY spectrum of **1**


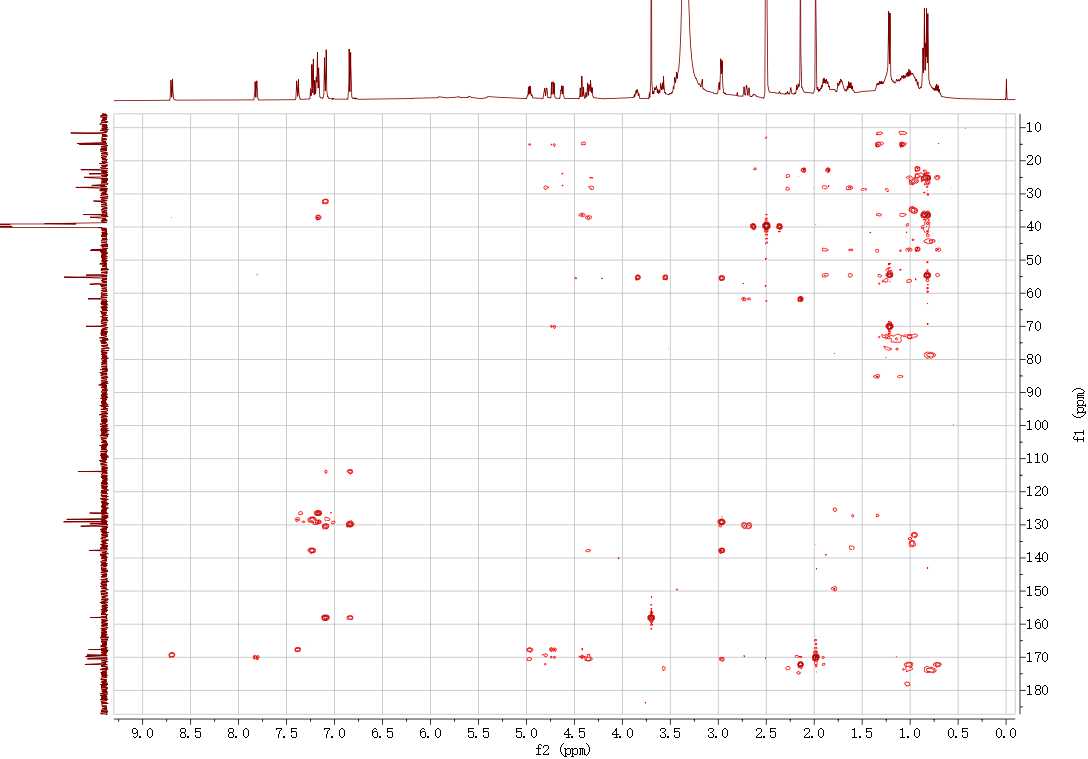


**Figure S5.** HMBC spectrum of **1**


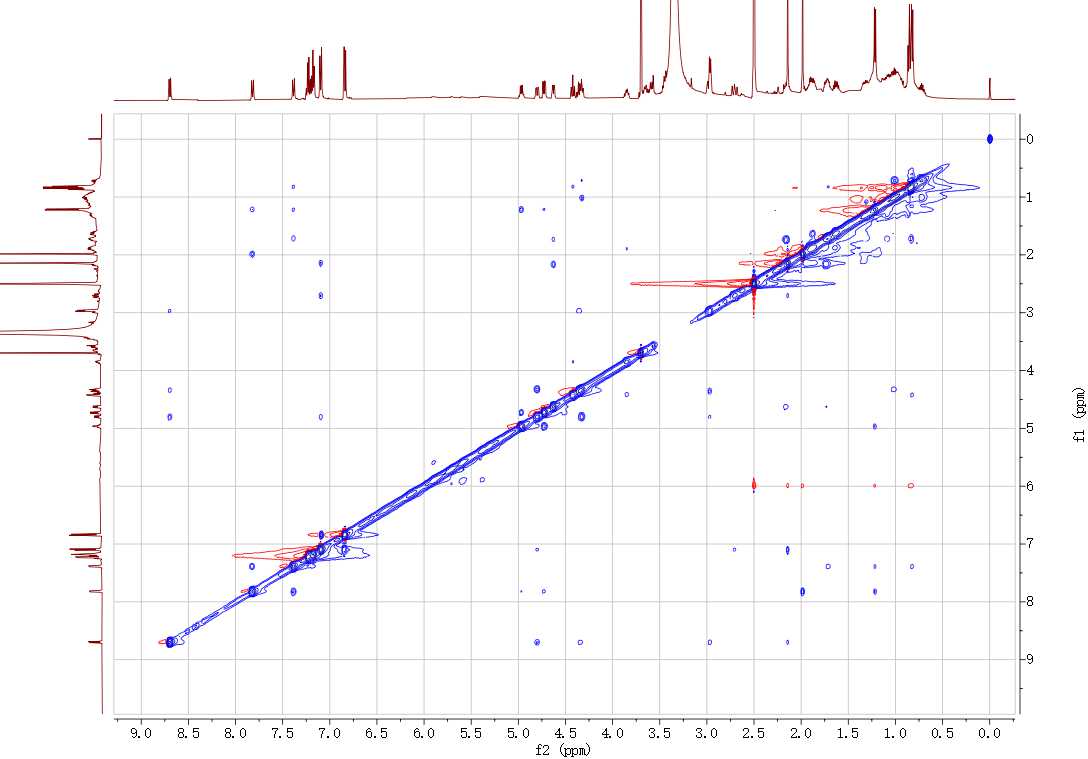


**Figure S6.** NOESY spectrum of **1**


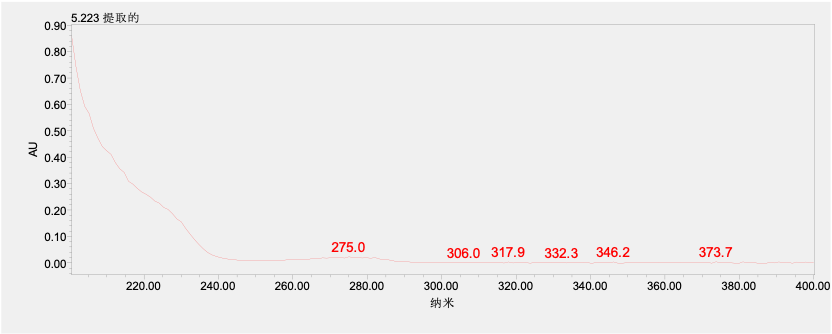


**Figure S7.** UV spectrum of **1** (100% MeOH/H_2_O, extracted from HPLC-DAD data)

**Figure S8.** HRESIMS spectrum of **1**

**
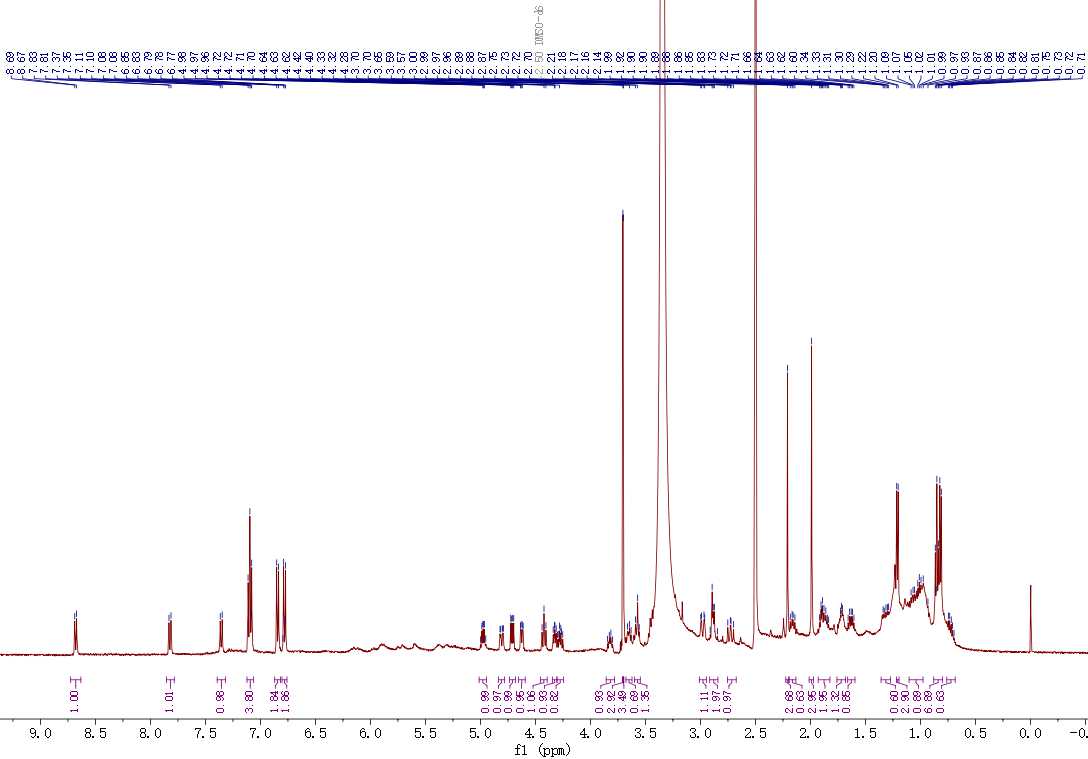
**

**Figure S9.** ^1^H NMR spectrum of **2** (DMSO-*d*_6_, 500 MHz)


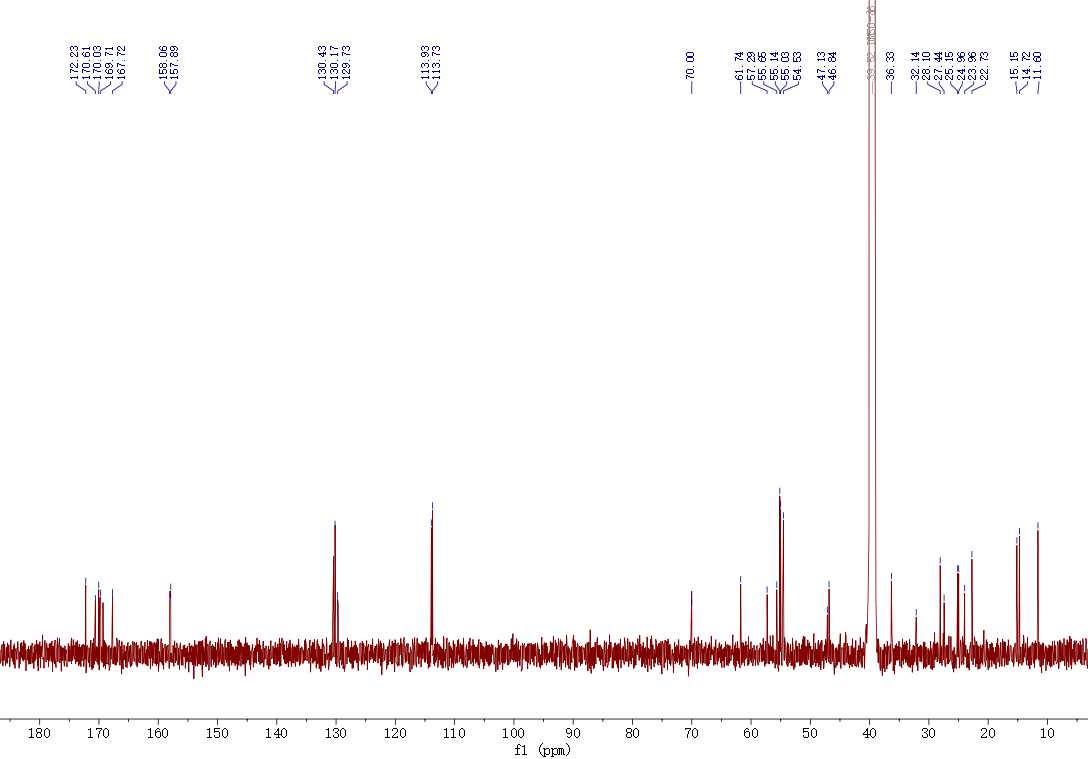


**Figure S10.** ^13^C NMR spectrum of **2** (DMSO-*d*_6_, 150 MHz)


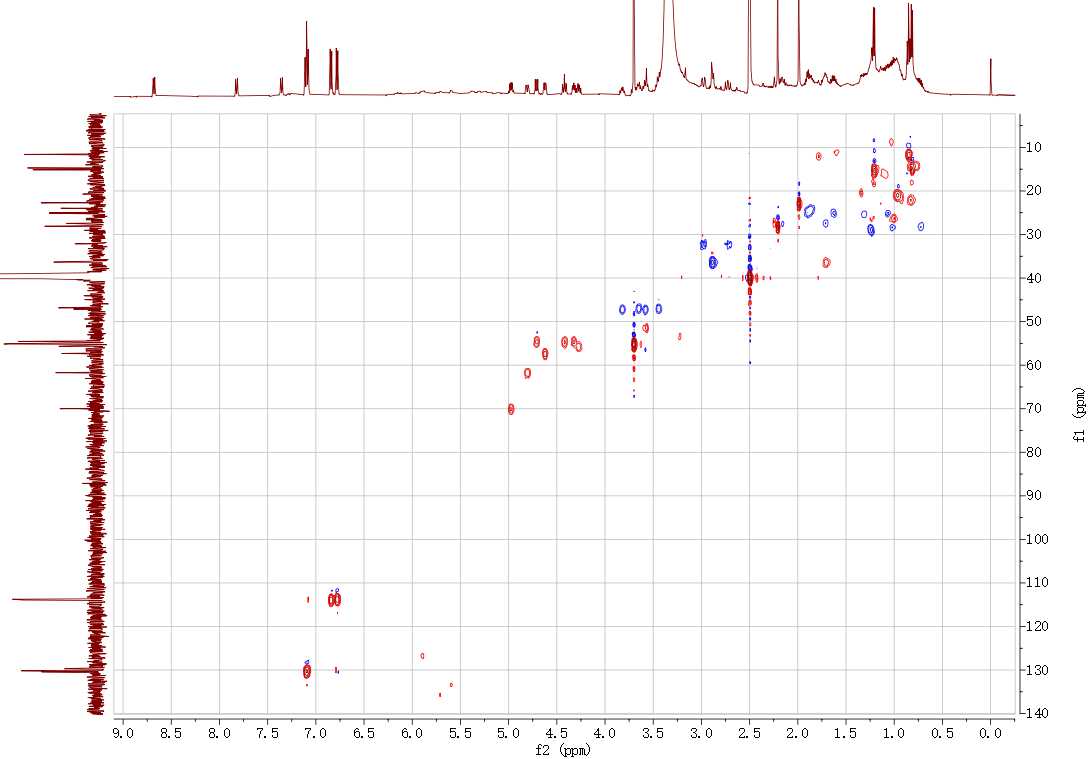


**Figure S11.** HSQC spectrum of **2**


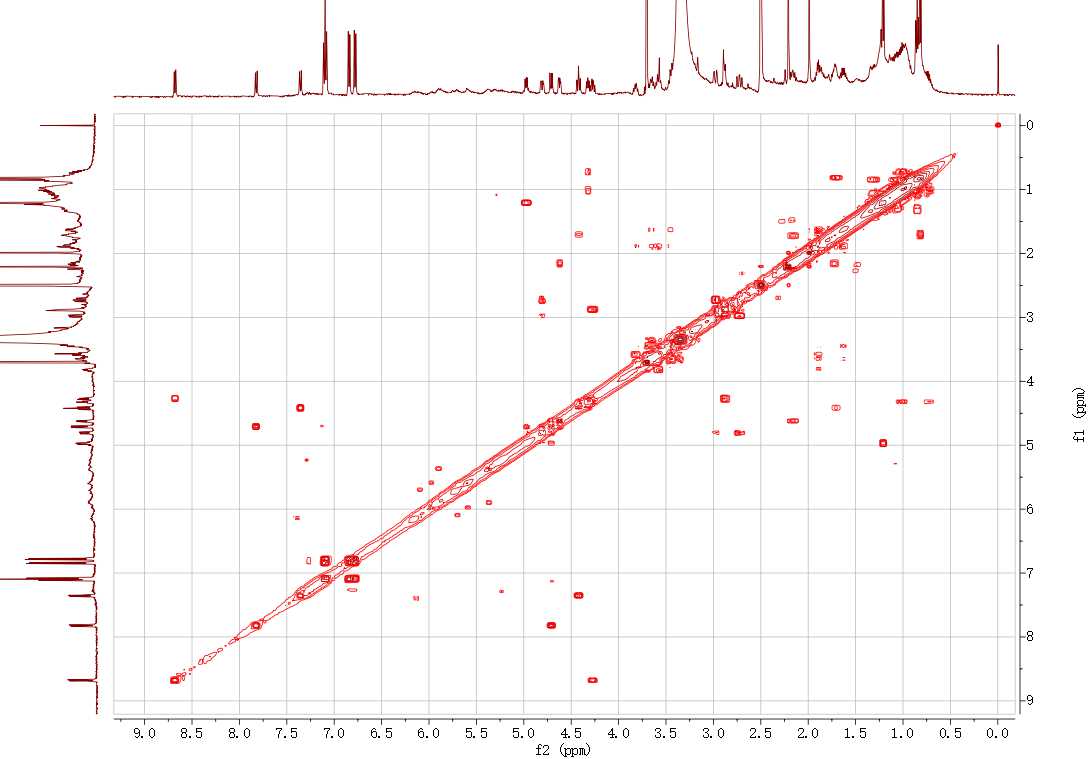


**Figure S12.** ^1^H-^1^H COSY spectrum of **2**


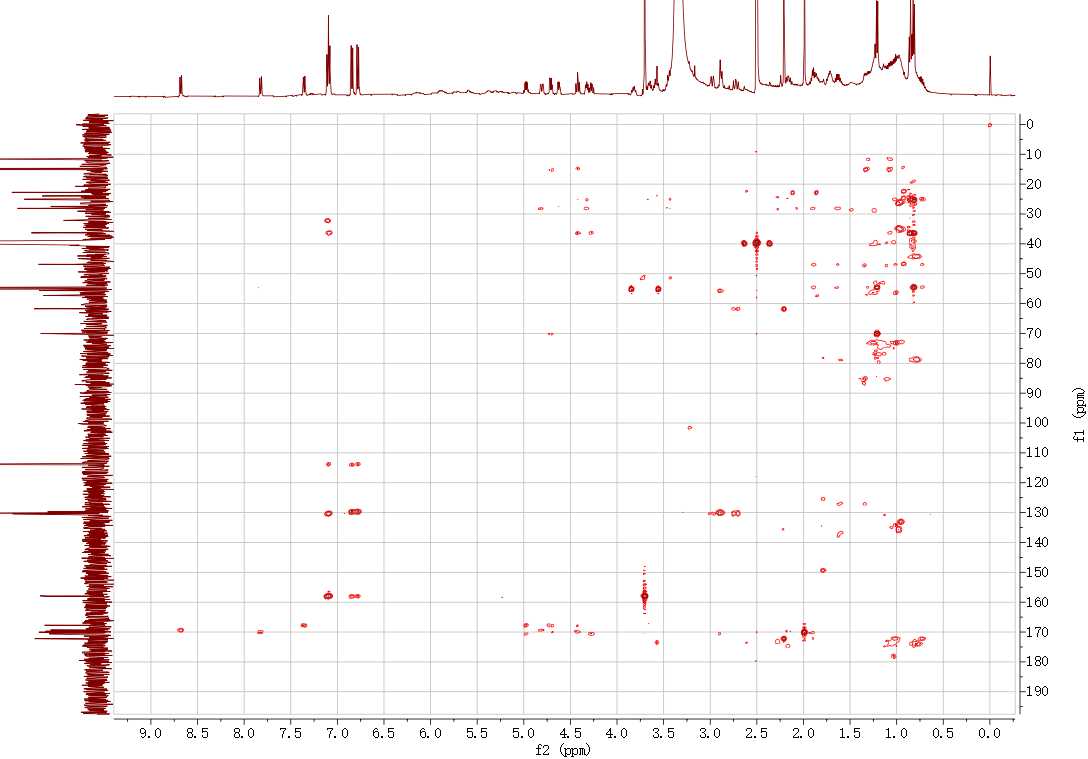


**Figure S13.** HMBC spectrum of **2**


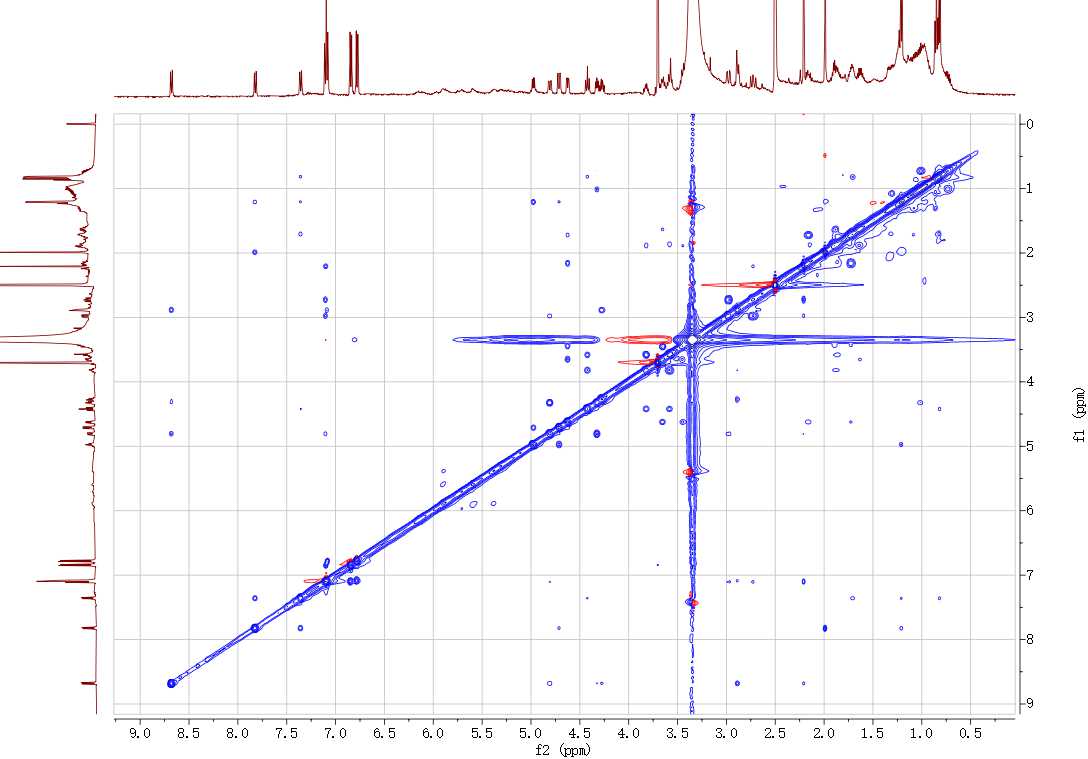


**Figure S14.** NOESY spectrum of **2**


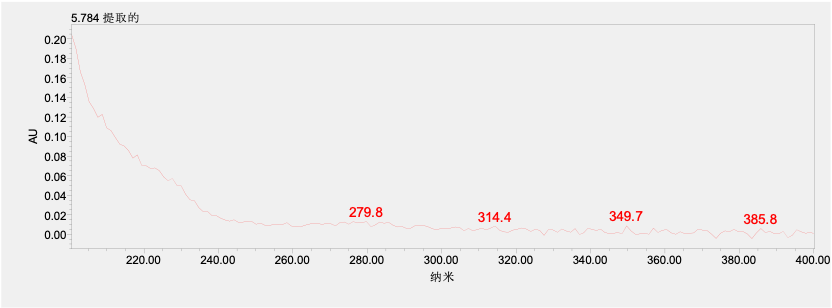


**Figure S15.** UV spectrum of **2** (100% MeOH/H_2_O, extracted from HPLC-DAD data)

**Figure S16.** HRESIMS spectrum of **2**

**
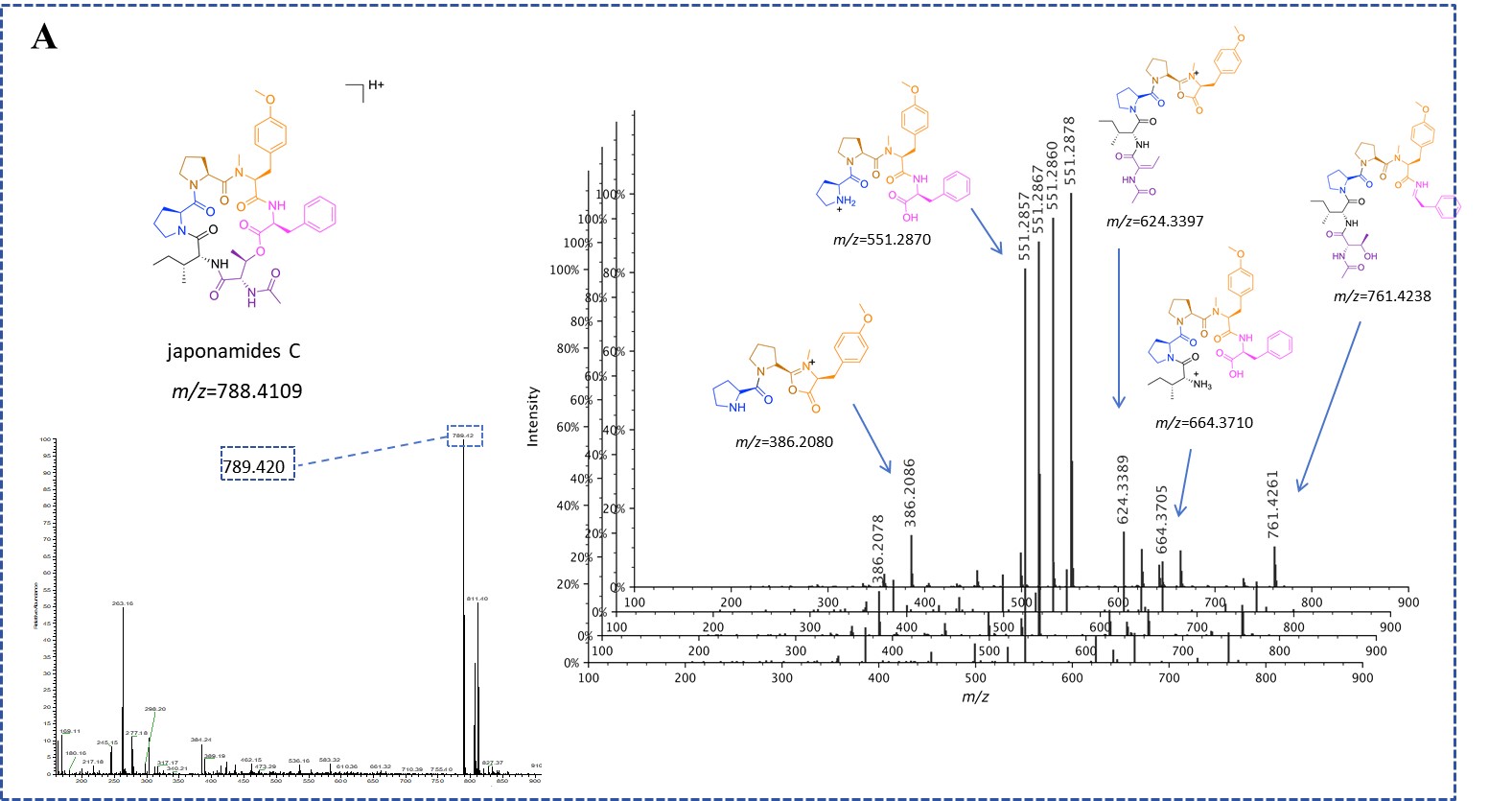
**

**
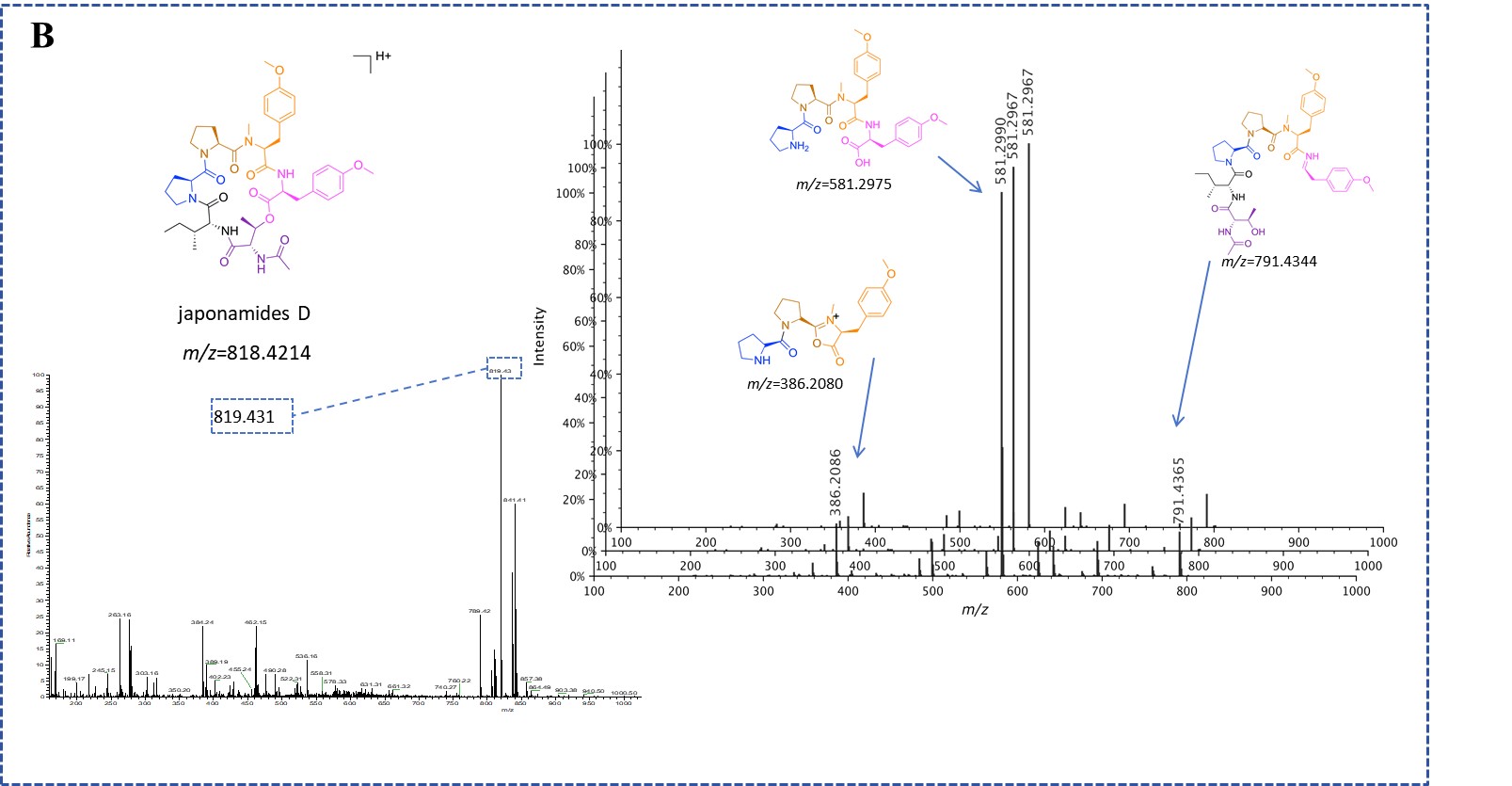
**

**Figure S17.** The tandem mass spectrometry of protonated ions at m/z 789.420 (**A**) and 819.431 (**B**), showing typical amino acid imine ions fragments of japonamides C (**1**) and D (**2**).
